# Effects of Transcranial Ultrasound Stimulation on Brain Amyloid Load in Alzheimer's Disease

**DOI:** 10.1002/alz70855_101923

**Published:** 2025-12-23

**Authors:** Jaeho Kim, Kyeonghwan Kim, Jeungeun Kum, YoungSoo Kim, Hyungmin Kim

**Affiliations:** ^1^ Dongtan Sacred Heart Hospital, Hallym University College of Medicine, Hwaseong‐si, Gyeonggi‐do, Korea, Republic of (South); ^2^ Yonsei University, Incheon, Incheon, Korea, Republic of (South); ^3^ Korea Institute of Science and Technology, Seoul, Korea, Republic of (South); ^4^ College of Pharmacy, Yonsei University, Incheon, Incheon, Korea, Republic of (South); ^5^ Korea Institute of Science and Technology, Seoul, Korea, Republic of (South)

## Abstract

**Background:**

The accumulation of β‐amyloid, the earliest pathological hallmark of Alzheimer's disease, contributes to neurodegenerative processes and cognitive decline. Recent studies highlight the potential of low‐intensity ultrasound, characterized by its safety and neuromodulatory effects, to enhance existing neuromodulation approaches, offering an innovative therapeutic avenue for Alzheimer's disease.

**Method:**

Focused ultrasound stimulation (FUS) was delivered to 5xFAD mice using implanted ultrasound transducers on their skulls. During a two‐week period, five 5xFAD mice underwent low‐intensity ultrasound stimulation while awake, with 30‐minute sessions administered five days per week. Blood Aβ42 levels and brain amyloid plaque loads were subsequently assessed and compared with control mice.

**Result:**

FUS treatment resulted in significant reductions in both the number and size of amyloid plaques, accompanied by an elevation in plasma Aβ42 levels. Importantly, no brain hemorrhages or significant alterations in GFAP levels were detected.

**Conclusion:**

Transcranial Ultrasound Stimulation demonstrates safety and efficacy in reducing brain amyloid load, underscoring its potential as a promising therapeutic strategy for Alzheimer's disease.

